# Linking electrical anomalies in the upper mantle to petrological and geochemical heterogeneities

**DOI:** 10.1093/nsr/nwag045

**Published:** 2026-01-22

**Authors:** Xiaozhi Yang, Yi-Gang Xu, Qun-Ke Xia

**Affiliations:** State Key Laboratory of Critical Earth Material Cycling and Mineral Deposits, School of Earth Sciences and Engineering, Nanjing University, Nanjing 210023, China; State Key Laboratory of Deep Earth Processes and Resources, Guangzhou Institute of Geochemistry, Chinese Academy of Sciences, Guangzhou 510640, China; Research Center for Earth and Planetary Material Sciences, School of Earth Sciences, Zhejiang University, Hangzhou 310027, China

**Keywords:** electrical anomalies, macroscale heterogeneities, upper mantle

## Abstract

The electrical structure of Earth’s interior, resolved by using geophysical surveys, is key to understanding its composition, dynamics and relevant properties. Electrically anomalous zones in the upper mantle have been frequently observed, yet the origin remains debated. The geophysically imaged electrical anomalies cannot be properly interpreted if the constraints on the mantle materials from petrological and geochemical surveys and mineral physics experiments are not combined. Studying mantle samples has revealed widespread heterogeneities in their mineral constituents, elemental compositions and thermodynamic properties, in addition to the local occurrence of melts and fluids. The heterogeneities are macroscale, ranging on the levels of meters to kilometers. Four conductive candidates have been identified for the electrical anomalies by using laboratory experiments under mantle conditions, including olivine owing to its oxidized state (but not water), lithologies (such as pyroxenites, eclogites and phlogopite-bearing assemblages due to enriched Fe, water and/or F), partial melt and aqueous fluids. Such materials are able to cause electrical anomalies in a variety of settings that are geophysically detectable, if connected forms, rational fractions and/or suitable temperature and redox states are spatially maintained along certain direction(s). Hydrous minerals except phlogopite (within their stability fields) and non-silicate minerals such as graphite, sulfides and carbonates are usually hard to produce mantle electrical anomalies. Mantle macroscale heterogeneities cause heterogeneous electrical structures. Geophysically imaged electrical anomalies in the upper mantle are intimately related to its petrological and geochemical evolution.

## INTRODUCTION

Of all the geophysical parameters (such as seismic velocity, density and magnetization), electrical conductivity is particularly sensitive to temperature and composition (especially subtle changes in Fe, water and other volatiles) of Earth materials, as well as the presence, character and fraction of melts and fluids in a rock matrix. Imaging of the electrical structure of the upper mantle offers crucial information on its structure, composition and properties. A high conductivity of 0.01–0.1 S/m to even greater (note that the uncertainty of geophysically yielded conductivity can be a factor of ≥∼2–3) or larger has been detected in many domains in both the continental [1–3] and oceanic [4–6] upper mantle. In active areas beneath mid-ocean ridges [7–9] and in mantle wedges [10–12], the conductivity is up to ∼0.3 to >1 S/m. The conductive zones are usually detected at depths of 40–150 km, though they extend to depths of >200 km in some continental regions [1,2] and they can be electrically anisotropic, with the conductivity varying by a factor of ∼10 along different directions [2,4,13].

The origin of the electrical anomalies has inspired many studies in past decades, but a general consensus has not been established. Several candidates have been proposed, such as the two popular suggestions of water in olivine (the most abundant mineral in the upper mantle) [14] and partial melt [15] and others of graphite [16] and sulfides [17] in grain boundaries. These models were initially conceived >20 years ago and later studies have led to some debates [18–21], as will be discussed below. On the other hand, the available models often assume a simple/ideal upper mantle for its constituents (e.g. peridotites dominated by olivine) and other properties including composition and temperature. The assumptions are not fully consistent with the petrological and geochemical studies of natural samples (e.g. rocks and basalts) showing that the upper mantle is heterogeneous from a variety of aspects [22–25], also partly supported by seismic evidence [26,27]. The origin of the electrical anomalies should be considered by taking into account these complexities.

Electrical conductivity is an intrinsic property of a material that is determined by its composition (for key charged species) and environmental conditions, being independent of its origin (e.g. locality and/or derived depth of a mantle sample). This is a fundamental basis for applying laboratory conductivity data of a sample (whether natural or synthetic) to Earth’s interior (and those of other planets). Recent mineral physics experiments have reported the conductivity of many upper mantle materials over a range of pressure, temperature, redox and composition (including water) conditions. The yielded data allow assessment of the roles of mantle heterogeneities in shaping electrical structures. We provide an integrated picture of macroscale heterogeneities and electrical anomalies in the upper mantle, mainly at depths of <200 km (especially in the oceanic asthenosphere: the definition differs between different studies and refers here to the shallow zone of high conductivity and low seismic velocity as widely treated [15,28,29]). We do not intend to comprehensively review the conductivity of a certain material(s) that has been done for several candidates [21,30] or the electrical anomalies through geophysical mappings (e.g. [4,6,15]). We first make a brief introduction of the two frequently adopted models of water in olivine and partial melt, by considering the debates and limitations from experimental studies. We then take a look at the macroscale heterogeneities in the upper mantle based on petrological and geochemical surveys. We finally compile the reported conductivity data of typical mantle materials and discuss the implications, by placing them in the context of the macroscale heterogeneities. We show that the mantle electrical structure is greatly influenced by the heterogeneities and four candidates are important for electrical anomalies in different settings.

## ON TWO CLASSIC CANDIDATES OF WATER IN OLIVINE AND PARTIAL MELT

Water as a hydroxyl in the olivine and partial melt of mantle rocks are two candidates that have been widely applied to explain mantle electrical anomalies. In the past two decades, many experiments have been carried out to test the two candidates. However, the reported data generated debates on and limitations to both of them. We assess the two candidates by using the conductivity data of typical experiments at high pressures. The compiled data are mainly measured by using impedance spectroscopy via wide-frequency sweeping that is necessary for Earth materials, because their resistance is frequency-dependent [31].

### Enhanced conductivity of olivine by water

**Figure 1.**
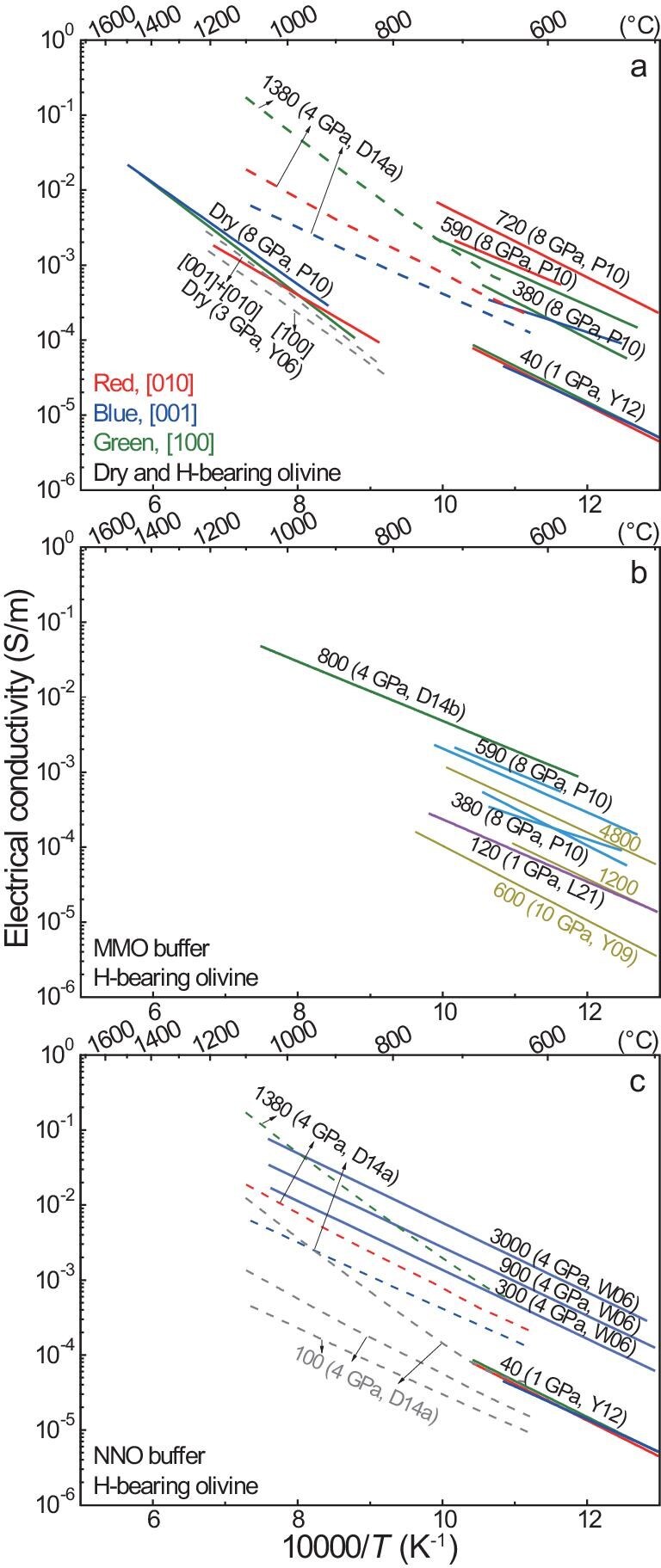
Electrical conductivity of water in olivine. (a) Single-crystal samples; (b, c) single-crystal plus polycrystalline samples with different redox buffers. Labels are water content (wt. ppm H_2_O) and other details. Shown in (a) is H-bearing olivine of the same or similar water contents along different orientations, with dry olivine [19,34]. Dashed grey lines in (a), Yoshino *et al.* [19] analyzed at 0.01 Hz; dashed grey lines in (c), data of Dai and Karato [38] scaled to 100 wt. ppm H_2_O by using a water content-conductivity equation [18] and a water-content exponent of ∼1 [122] for H conduction in nominally anhydrous minerals. Mg# is ∼90 in all olivines. All water contents are based on Bell *et al.* [39]. Under the given conditions, conductivity increases with increasing water content, and orientation-related electrical anisotropy is insignificant for both dry and H-bearing olivines (except in some reports: see text). MMO, Mo–MoO_2_; NNO, Ni–NiO. Data sources: W06, Wang *et al.* [18]; Y06, Yoshino *et al.* [19]; Y09, Yoshino *et al.* [33]; P10, Poe *et al.* [34]; Y12, Yang [35]; D14a, Dai and Karato [38]; D14b, Dai and Karato [36].

Water was initially suggested in 1990 to enhance olivine conductivity [14] and was later proposed to be responsible for mantle electrical anisotropy owing to the lattice-preferred orientation of olivine [4,32]. It was not until 2006 that experimental data on the conductivity of H-bearing olivine started to be reported [18,19,33–38]. A widely accepted data trend is that, under given conditions, the conductivity increases with the water content (for olivine and other nominally anhydrous minerals). Typical data are plotted in Fig. 1 (note that the analyses of Yoshino *et al.* [19] were conducted at 0.01 Hz, which recorded electrode effects but no lattice conduction [31], and the water-rich samples of Poe *et al.* [34], with low activation enthalpy (30–50 kJ/mol), were likely subject to dehydration, so their data are accordingly excluded). For convenience, the water contents of both laboratory and natural olivines are presented based on the Bell *et al.* [39] calibration, e.g. a factor of 3 is applied to the Paterson [40] method in previous studies [18,33,36,38] and the general results are unaffected by the calibration.

Most conductivity experiments on H-bearing olivine are conducted at below ∼700°C so that dehydration upon heating is minimized. For single-crystal olivine with 40–590 wt. ppm H_2_O, the electrical anisotropy is negligible under given conditions [34,35] (Fig. 1a). For olivine with ∼1380 wt. ppm H_2_O, the electrical anisotropy is profound [38]: it is enhanced toward 1100°C ([100] the highest and [001] the lowest) and is larger at low temperatures (<700°C) than that of olivine with 40–590 wt. ppm H_2_O (Fig. 1a). It is likely that the conductivity of highly water-enriched olivine is anisotropic and the extent of the anisotropy is temperature-sensitive. However, water-rich conditions are unexpected for olivine: the maximum water solubility in the entire upper mantle is only ∼800 wt. ppm H_2_O at well-controlled pressure, temperature, redox state, mineral chemistry and coexisting phases [41] and the water content of most natural samples is <100 wt. ppm H_2_O at depths of <200 km (see later). Thus, the electrical anisotropy of olivine in the upper mantle, of low-to-moderate water contents, should be insignificant. The conductivity data of olivine with 40–590 wt. ppm H_2_O [34,35] are consistent for the expected role of water, but the data of water-rich olivine with ∼1380 wt. ppm H_2_O [38] deviate from the trend at low temperatures (Fig. 1a).

The weak electrical anisotropy of H-bearing olivine allows direct comparison of the conductivity of single-crystal and polycrystalline samples under similar conditions (note that the pressure effect is small but measurable for dry and H-bearing olivines, e.g. the conductivity is reduced by 0.1–0.2 log units for an increase in pressure of 2–3 GPa [37,42,43]). The compiled data are grouped by redox conditions, as Mo–MoO_2_ (MMO: Fig. 1b) and Ni–NiO (NNO, which is ∼3–4 log units more oxidized than MMO: Fig. 1c). Under MMO-buffered conditions, the conductivity increases with water content from ∼120 to 380 to 590 to 800 wt. ppm H_2_O [34,36,37] and, under NNO-buffered conditions, the conductivity increases with water content from ∼40 to 300 to 900 to 3000 wt. ppm H_2_O [18,37]. The two most significant exceptions appear in the results of Yoshino *et al.* [33] (who reported inconsistent water contents in wt.% and ppm H/Si units [44] and our re-estimated values are based on their contents in wt.% units) and Dai and Karato [38]: in both reports, the conductivity is too low for water contents of 600–4800 wt. ppm H_2_O and is inconsistent with the expected trend regarding the role of water shown in other studies (Fig. 1b and c).

The activation enthalpy of H conduction in olivine is 80–90 kJ/mol in most studies at <1000°C [18,33–37], except the 30–50 kJ/mol of likely dehydrated water-rich samples in Poe *et al.* [34] and 90–140 kJ/mol of water-rich samples in Dai and Karato [38]. This implies that, for H conduction, the activation enthalpy is probably independent of the olivine water content at low-to-moderate concentrations. For the water contents in the mantle at depths of <200 km (<500 wt. ppm H_2_O from solubility experiments [41,45,46] and <100 wt. ppm H_2_O in most natural samples: see below), the conductivity of olivine by extrapolating H conduction to mantle temperatures of 1000–1300°C is commonly <0.01 S/m, despite the increase at higher water contents (Fig. 1b and c). This has also been predicted by Gardés *et al.* [44] through compiling previously reported data (without filtering/grouping the data for dehydration, single frequency analyses and/or redox controls). If scaled to <100 wt. ppm H_2_O as in most mantle samples, the conductivity of olivine with ∼1380 wt. ppm H_2_O in Dai and Karato [38] is also <0.01 S/m at 1000–1300°C (Fig. 1c). Bulk conductivity by both small-polaron and H conduction will be discussed later.

### Enhanced bulk conductivity by partial melt

Partial melt was first suggested in the 1970s to interpret mantle electrical anomalies, shortly after the mantle low-velocity zone due to ∼1 vol.% melt was hypothesized [47,48]. Two often considered candidates are basaltic melts and carbonatite melts. Early attention was largely paid to dry basalts, of which the required melt fraction for the observed high conductivity reaches up to 5–15 vol.% or even larger at an assumed temperature of 1100–1300°C [15]. Subsequently, the roles of H_2_O and CO_2_, and also carbonatites, in affecting melt conductivity were considered and the needed melt fraction was reduced.

**Figure 2.**
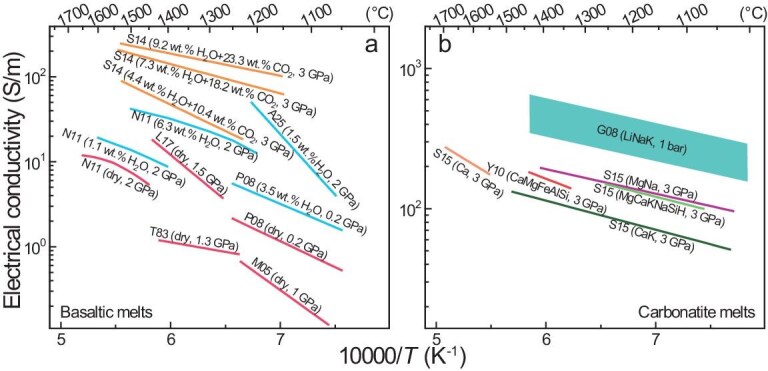
Electrical conductivity of melts. (a) Basaltic melt; (b) carbonatite melt. Sample details are labeled. Conductivity of carbonatites and H_2_O- and CO_2_-rich basalts is higher than those of both dry and water-poor basalts, and data are not consistent under comparable conditions for either dry or H_2_O–basalts (see text). Data sources: T83, Tyburczy and Waff [49]; M05, Maumus *et al.* [50]; G08, Gaillard *et al.* [56]; P08, Pommier *et al.* [51] (sample of VS88-65B); Y10, Yoshino *et al.* [58]; N11, Ni *et al.* [52]; S14, Sifré *et al.* [28]; S15, Sifré *et al.* [59]; A25, Ashley *et al.* [53]. Abbreviations in (b): LiNaK, Li_2_CO_3_, Na_2_CO_3_, K_2_CO_3_ and/or CaCO_3_; MgNa, MgCO_3_ and Na_2_CO_3_; MgCaKNaSiH, MgCO_3_, CaCO_3_, K_2_CO_3_, Na_2_CO_3_ and SiO_2_ with ∼8 wt.% H_2_O; Ca, CaCO_3_; CaK, CaCO_3_ and K_2_CO_3_.

The conductivities of dry and H_2_O-/CO_2_-bearing basaltic melts are given in Fig. 2a. For both dry and H_2_O-bearing basalts under comparable conditions, the reported data [49–54] are inconsistent, varying by a factor of >10. This may be due to different sample chemistry and technical issues (at high pressures, small changes in pressure of 1–2 GPa only weakly affect melt conductivity [54]). For instance, the sample Na (key to melt conductivity [55]) and Fe contents differed, and Tyburczy and Waff [49] analysed samples at 2000 Hz while others used frequency sweeping. For dry basalts, the conductivity is ∼1–10 S/m at 1100–1300°C (0.2–2 GPa) [49–54]. For H_2_O-bearing basalts, the conductivity is enhanced and is 3–30 S/m for the contents of ∼1–6 wt.% H_2_O at 1100–1300°C (0.2–2 GPa) [51–53]. The enhancement is probably due not to water, but to the enhanced mobility of Na under more hydrous conditions [51,55]. For H_2_O- and CO_2_-rich basalts (or carbonated basalts), the conductivity is 20–200 S/m for contents of ∼4–9 wt.% H_2_O and 10–23 wt.% CO_2_ at 1100–1300°C (3 GPa) [28], possibly indicating their even greater Na mobility.

The conductivities of carbonatite melts are provided in Fig. 2b. High values of 200–550 S/m at 1100–1300°C (1 bar) were reported for LiNaK (Li_2_CO_3_–Na_2_CO_3_–K_2_CO_3_) and Na-/K-rich (Li_2_CO_3_–Na_2_CO_3_–K_2_CO_3_–CaCO_3_) carbonatites, which were suggested to lead to mantle high conductivity [56]. However, carbonatites in the mantle are commonly rich in Mg and Ca, with variable amounts of other species such as Fe, Si and Al, and are poor in highly mobile species such as Li, Na and K [57]. Mantle carbonatites and their analogs with various compositions are less conductive [58,59]: the conductivity is 60–200 S/m at 1100–1300°C and 3 GPa, similar to those of H_2_O- and CO_2_-rich (carbonated) basalts (Fig. 2). The activation enthalpy of carbonatite melts is usually 30–60 kJ/mol, except for CaCO_3_ melt (∼75 kJ/mol) [56,58,59], and is usually lower than that of dry H_2_O-/CO_2_-bearing basaltic melts, e.g., 60–110 kJ/mol [49,51,52,59] (note that the high activation enthalpy of 180–200 kJ/mol was reported for dry basalts by Maumus *et al.* [50] and Laumonier *et al.* [54]).

Given the conductivity of a melt and olivine, the required fraction of melt for high conductivity can be estimated by using a mixing model, e.g. the parallel solution or the Hashin–Shtrikman upper bound [54]. This has been routinely applied in melt-conductivity studies [15,28,49–52,56,59], though there are studies on melt–olivine mixtures in which a mixing model is still used [54,58]. The melt fraction needed for the conductivity of 0.01–0.1 S/m is ∼0.5–3 vol.% for basaltic melts [52,54,58] and 0.03–0.5 vol.% for carbonatite melts or carbonated basaltic melts [28,56,58]. Basaltic melts rather than carbonatite melts can be present in the shallow mantle [60] and highly H_2_O- and CO_2_-rich basalts are not always compatible with studies of mid-ocean ridge basalts (MORB) [61]. Hence, basaltic melts of 0.5–3 vol.% are more often used to explain the electrical anomalies for studies of both mineral physics [52,54,58] and electromagnetic mappings [5,9,62]. Available studies and mixing models usually assume ideal mixing for the bulk conductivity of a melt–mineral system, e.g. complete grain wetting by the melt (e.g. 0° dihedral angle), all melt comprising connected films/tubes and the absence of grain-size effects. The assumptions are unrealistic for melts in the shallow mantle [63,64] and the inferred melt fraction under fixed conditions by ideal mixing can be underestimated by a factor of ∼5–10 or even greater [65]. The value of 0.5–3 vol.% should actually be several to >10 vol.%. This raises open questions, especially for the asthenosphere: (i) is the large fraction of melt stable? (ii) if the conductive structure is not transient, then how can the melt be effectively trapped? (iii) the petrological and physical grounds favor <1 vol.% and mostly <0.1 vol.% melt in the shallow mantle away from ridges [60,66] and those values are lower than the required melt fractions and (iv) in the asthenosphere, the melt fraction of several vol.% to higher would reduce the seismic velocity more substantially than that expected for the low-velocity zone by ∼1 vol.% melt [48,67].

## MANTLE HETEROGENEITIES REVEALED BY PETROLOGICAL AND GEOCHEMICAL STUDIES

The upper mantle is in general subsolidus, though melts and fluids can be regionally present. Many samples such as xenoliths, massifs, abyssal rock patches, diamond-hosted mineral inclusions and MORB provide a window into the mantle. Samples have originated mostly from the lithospheric mantle, but MORB were due to melting of the asthenospheric mantle. Relevant studies have demonstrated widespread heterogeneities from lithological, compositional and thermodynamic aspects (note that MORB studies offer indirect constraints for the asthenosphere) [22–25]. The heterogeneities are macroscale, from meters to kilometers (supported by seismic imaging [26,27]: melts or fluids in the regional upper mantle also reflects its heterogeneities). The heterogeneities have been well documented in the continental regions due to the better accessibility of mantle samples and they are also expected in the oceanic regions, including the asthenosphere [22–25]. The heterogeneities contrast with the widely assumed simple/ideal mantle in mineral physics and geophysical studies on the electrical anomalies. The discussions below focus on solid materials, based mainly on xenoliths that have better preserved the source information, but the general results hold for both continental and oceanic shallow mantle.

### Lithological heterogeneity

The upper mantle is dominated by peridotites, consisting of olivine, orthopyroxene, clinopyroxene and spinel/garnet—olivine is up to ∼95 vol.% in dunites and this greatly determines the bulk conductivity [18,19,34,68]. Besides peridotites, many other rocks (and/or mineral assemblages) are widespread, being locally enriched (Table 1). The most typical constituents are pyroxenites and eclogites, as well as rocks bearing hydrous minerals (e.g. phlogopite and amphibole) and non-silicate minerals (e.g. graphite and sulfides). Multiple origins are involved for these components, such as modal metasomatism.

**Table 1.** Major rock types in the upper mantle observed from mantle xenoliths.

Type	Characteristics	Typical examples (locality)
**(A) Cratonic/circum-cratonic xenoliths erupted by Kimberlite-related volcanics**		
A1: Coarse Mg-rich, low-T peridotites	Often abundant. Mostly harzburgites and lherzolites: equilibration *T* < 1100°C and equilibration *P* from ∼2 to >6 GPa	N. Lesotho; Kaapvaal craton; Siberia; Jericho, Slave craton
A2: Coarse, Fe-rich low-T peridotites and pyroxenites	Widespread, locally abundant. Mainly garnet lherzolites and garnet websterites, but also clinopyroxenites and orthopyroxenites	Kaapvaal craton; Jericho, Slave craton; Mzongwana, Kaapvaal craton
A3: Dunites	Widespread, locally common. Coarse to ultra-coarse olivine dunites, and often fine- to medium-grained more Fe-rich dunites	Siberia, notably Udachnaya
A4: Deformed low-T peridotites and pyroxenites	Widespread, locally common. Porphyroclastic or mosaic porphyroclastic textures. Equilibration *P*–*T* similar to those of type AI	Jericho, Slave craton
A5: Deformed high-T peridotites	Widespread but variable abundance. Equilibration *T* from ∼1100°C to >1500°C and equilibration *P* generally 4.5 to >6.5 GPa	N. Lesotho; Jagersfontein, Kaapvaal craton; Siberia; Slave; Somerset Island
A6: Phlogopite-rich mafic mantle xenoliths	Widespread and locally common. Two main suites: (i) MARID suite (mica–amphibole–rutile–ilmenite–diopside); (ii) PIC suite (phlogopite–ilmenite–clinopyroxene)	Kimberley
A7: Pyroxenite sheets rich in Fe and Ti	Locally present. Orthopyroxene and clinopyroxene-rich rocks with widely variable olivine and garnet, often with phlogopite	Matsoku
A8: Modally metasomatized peridotites	Widespread, variable abundance. Two main groups: phlogopite peridotites and phlogopite-K-richterite peridotites	Matsoku; Kimberley pipes; Jagersfontein
A9: Eclogites, grospydites, alkremites and variants	Very widespread, rare to locally abundant. High Cr, Ca, Fe and Mn in omphacite. Garnet can be Na- and Mg-rich or Na-poor	Roberts Victor; Jagersfontein; Orapa; Kaapvaal craton; Udachnaya, Siberia
A10: Megacrysts	Widespread and locally present, garnets, clino- and orthopyroxenes and phlogopite most common	N. Lesotho; Monastery; Jagersfontein; Kaapvaal craton; ocean plateau; Colorado
A11: Polymict aggregates	Polymict aggregates of peridotite, eclogite and megacrysts, of variable grain size, some containing quenched melt	Bultfontein, De Beers and Premier mines, Kaapvaal; Malaita
A12: Diamond and inclusions in diamonds	Widespread and closely related to cratons. Inclusion suites divided into peridotitic and eclogitic parageneses	All cratons
**(B) Non-cratonic xenoliths erupted by alkalic and potassic mafic magmas**		
B1: Cr-diopside lherzolite group	Very widespread and common. Mainly spinel-facies but can be garnet-bearing. Include harzburgites, orthopyroxenites, clinopyroxenites, websterites and wehrlites	Victoria, Australia; Vitim; San Carlos; Eifel; Hawaii; Scotland; Thumb; Pali-Aike
B2: Al-augite wehrlite pyroxenite group	Widespread and common. Often clinopyroxene-rich rocks, but widely variable: wehrlites, clinopyroxenites, dunites, websterites, lherzolites	Victoria, SE Australia; San Carlos and other W. USA localities; Hawaii
B3: Garnet pyroxenite group	Widespread but not abundant. Coarse-grained, undeformed textures, sometimes layered. Basaltic bulk compositions	Delegate, SE Australia; Salt Lake Crater, Hawaii
B4: Modal metasomatic group	Widespread. Wehrlite-clinopyroxenites with mica, glimmerites. Silicate glass by melting of amphibole, clinopyroxene or phlogopite common	Nunivak, Alaska; SE Australia; Vitim; Loch Roag and Fife, Scotland
B5: Megacrysts	Widespread with variable abundance. Usually large single crystals, including Al-augite, Al-bronzite, olivine, kaersutite and pyrope	SE Australia; Loch Roag, Scotland

Classifications of xenoliths and their characteristics are based on Pearson *et al.* [84]. Samples are mainly from continental regions (e.g. lithospheric mantle), but similar results are expected for oceanic upper mantle (e.g. lithospheric mantle plus shallow asthenospheric mantle).

In the upper mantle, pyroxenites are linked to high pressure cumulating in magmas, the recycling of oceanic crusts or the metasomatism of peridotites [69–71] and eclogites are due to recycled oceanic crusts [72], continental crust delamination [73] or cumulation at high pressures [74]. Pyroxenites mainly consist of pyroxenes, with/without minerals including olivine, and eclogites chiefly contain omphacite and garnet, with accessory minerals such as rutile. Statistical analyses of mantle xenoliths show the local fractions of up to >10 vol.%, e.g. ∼20 vol.% for pyroxenites in Jericho Kimberlite, Canada [75] and 15–25 vol.% for eclogites in Roberts Victor Kimberlite, South Africa [76] and Jericho Kimberlite, Canada [77]. While these proportions may be subject to biased sampling and preserved xenoliths during entrainments by magmas, they still reflect the local enrichment of those rocks (note that, in some peridotite massifs, the pyroxenite fraction is up to ∼10 vol.% [70]). Pyroxenites and eclogites usually occur as veins, dykes and/or layers that are elongated, aligned and/or twisted in peridotites, e.g. by mantle convection and deformation, and their occurrences led to the marble cake model [22]. Isotopic studies of MORB and abyssal peridotites (the residues after MORB extraction) also favor the presence of pyroxenites and eclogites in the asthenosphere [23,25].

Hydrous minerals such as amphibole, serpentine and phlogopite are often restricted to subduction zones, though amphibole and especially phlogopite can occur in the normal mantle away from subduction zones [78–80]. The exact amount of hydrous minerals in subducting slabs (and mantle wedges) is unknown but can be very low [79,81]. In general, hydrous minerals dehydrate significantly when subducting to depths of >90 km [82]. In the normal mantle, phlogopite is usually due to metasomatism, through peridotite reactions with melts or fluids, and is widespread and locally enriched (on % levels) [83,84]. In the PIC (phlogopite–ilmenite–clinopyroxene) and MARID (mica–amphibole–rutile–ilmenite–diopside) xenolith suites, phlogopite can be >50 vol.% [84]. Phlogopite of metasomatic origin often occurs as veins, but the connection can break during sample exhumation [85]. Amphibole is also frequently found as a metasomatic product in mantle xenoliths, though some samples might be of igneous origin [86]. Differently from phlogopite that is stable to depths beyond ∼250 km, amphibole is usually stable over a narrow depth range of 40–80 km and is unstable in most regions in typical continental and oceanic shallow mantle [87]. Non-silicate minerals of graphite, sulfides and carbonates (e.g. dolomite and magnesite) are seen in mantle xenoliths and they may form through metasomatism or immiscibility [88–91]. When present, these minerals occur chiefly in dispersed forms, e.g. between grains or enclosed in primary minerals, and the fractions are usually very low, e.g. <0.1% [92]. Other minerals, such as zircon, rutile and diamond, are unable to greatly affect the electrical properties of the mantle (owing to their isolated occurrences and extremely low amounts) and so they are not discussed.

### Compositional heterogeneity

The bulk composition and conductivity of a rock system are controlled by its mineral constituents. The composition of a mineral, especially for critical charged species, is key to its conductivity under given conditions. The two most critical charged species of minerals in the upper mantle are Fe-related small polarons and protons (H). They are important to electrical conduction in dry and H-bearing olivine (and pyroxenes + garnet), respectively, e.g. small-polaron versus H conduction [18,19,33–38]. Other species are critical for ionic conduction, e.g. F in phlogopite [93,94] and Mg vacancies in olivine (important at >1400°C) [33]. The conductivity is often enhanced at a greater content of the charged species (but H conduction in olivine differs [34,37,44]: see later). The heterogeneities of Fe and water in the main minerals, for the same type of rock and between different rocks, are discussed.

**Figure 3.**
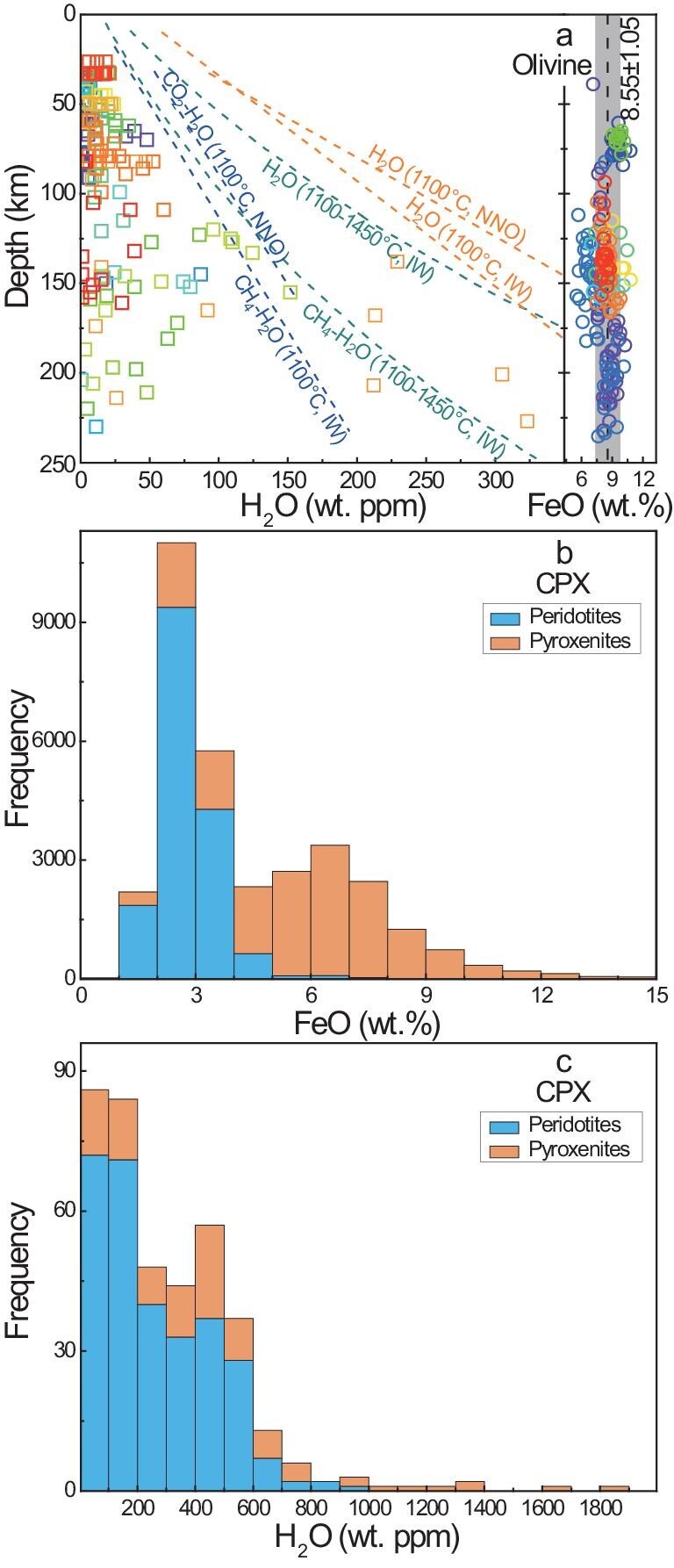
Fe and water contents of typical minerals in mantle xenoliths. (a) Fe and H_2_O of olivine in peridotites; (b) Fe and (c) H_2_O of clinopyroxene in peridotites and pyroxenites. Dashed lines in (a) indicate the water solubility of olivine at well-controlled pressure, temperature, redox state, silica activity and mineral chemistry, and coexisting minerals and fluids for the mantle (fits at 1100°C or over 1100–1450°C). Water data in (a) are based on Bell *et al.* [39] (making data comparison between natural samples, solubility runs and conductivity runs meaningful): squares, mantle xenoliths [95]; dashed lines, solubility fits [41,45,46]. FeO data in (a) are for peridotite xenoliths compiled by Zhang *et al.* [41]: dashed line indicates average value and shaded area indicates deviation (8.55 ± 1.05 wt.%). Different symbols in (a) are for different regions, data in (b) are from the GEOROC database [96], and data in (c) are for peridotites [95] and pyroxenites (water contents in (c) are from [98–100], measured mostly by using Fourier-transform infrared (FTIR) analyses and some by vacuum extraction and ion microprobe).

For olivine in garnet peridotite xenoliths (as compiled in Zhang *et al.* [41]), the FeO content (total Fe) is mostly ∼6.5–10.5 wt.%, with an average of ∼8.6 ± 1.1 wt.%, and the depth variation of FeO is not profound (Fig. 3a). The Mg# value (= 100 × Mg/(Mg + Fe), in molar ratios) is mostly ∼90, observed also for olivine in spinel peridotite xenoliths (based on the compilation of Demouchy and Bolfan-Nasanova [95]). In both garnet- and spinel-bearing peridotite xenoliths, the FeO content clusters at ∼4–8 wt.% in orthopyroxene, 1–5 wt.% in clinopyroxene and 6–10 wt.% in garnet (see Fig. 3b for clinopyroxene as an example [96]). The FeO contents of orthopyroxene, clinopyroxene and garnet in both pyroxenite and eclogite xenoliths are, however, relatively high, each exhibiting a wide variation range. Typically, the FeO content is mainly ∼6–20 wt.% for orthopyroxene in pyroxenite xenoliths, 2–14 wt.% for clinopyroxene in pyroxenite and eclogite xenoliths and 8–32 wt.% for garnet in eclogite xenoliths (see Fig. 3b for clinopyroxene as an example [96]). Note that, although they belong to the same mineral group, Fe-bearing diopside in peridotites and pyroxenites differs chemically from omphacite in eclogites, e.g. for Na and other species.

Water (more accurately H) is only a minor species in mantle olivine, orthopyroxene, clinopyroxene and garnet. In each mineral, the water content varies substantially between samples and localities. For olivine in peridotite xenoliths, the water content is mostly <100 wt. ppm H_2_O at depths of <200 km (Fig. 3a). For the asthenosphere (MORB sources), the bulk water content is typically 50–150 wt. ppm H_2_O (note that enriched sources of MORB with >300 wt. ppm H_2_O represent a minor portion of the convecting mantle) [61], leading to olivine water content of mostly <50 wt. ppm H_2_O. The contents are less than the water solubility of olivine under water-saturated and well-controlled mantle conditions, of which the maximum is <500 wt. ppm H_2_O at depths of <200 km [41,45,46] (Fig. 3a). This suggests that the upper mantle is in general water undersaturated. For other minerals in peridotite xenoliths, the water contents are mostly <5 to ∼600 wt. ppm H_2_O in clinopyroxene, <5 to ∼400 wt. ppm H_2_O in orthopyroxene and <5 to ∼100 wt. ppm H_2_O in garnet [95,97]. In pyroxenite xenoliths (including pyroxene megacrysts), the water contents are often <5 to 1800 wt. ppm H_2_O in clinopyroxene and <5 to ∼400 wt. ppm H_2_O in orthopyroxene [98–100], of which the former values can be higher than those in peridotite xenoliths (despite data overlapping: Fig. 3c) and the latter values resemble those in peridotite xenoliths. In eclogite xenoliths, the water contents are up to ∼1900 wt. ppm H_2_O in omphacite (among the most water-rich nominally anhydrous minerals) and 900 (mostly <300) wt. ppm H_2_O in garnet [101,102], being greater than in clinopyroxene and garnet in peridotite xenoliths.

### Thermal and redox heterogeneity

Pressure, temperature and redox state (quantified by oxygen fugacity, *f*O_2_) are three key thermodynamic parameters. The latter two greatly affect the conductivity of mantle minerals. With increasing pressure (depth) in the mantle, the temperature increases and the redox state becomes more reduced. These are the general radial trends for a simple, ideal Earth. However, the thermal and redox properties are complicated in the realistic upper mantle and are highly heterogeneous along lateral directions and on different scales.

**Figure 4.**
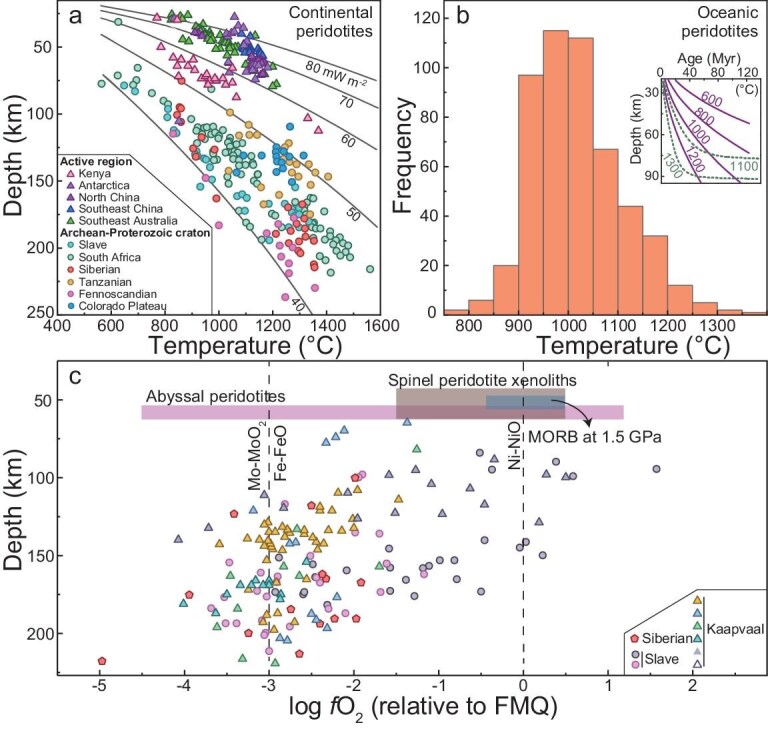
Thermodynamic properties of the upper mantle. (a) Temperature of continental peridotite xenoliths, (b) temperature of abyssal peridotites and (c) redox state of mantle samples. Temperature obtained by using the routine four-phase thermometry barometry [106,107] for garnet peridotites in (a) and two-pyroxene thermometry [111] for spinel peridotites in (b) (similar temperature patterns as in (b) are obtained by using two-pyroxene thermometry [106] at an assumed pressure of 1.5 GPa). In (a), xenolith data and heat flux, from Lee *et al.* [104] and Artemieva [105]; in (b), abyssal peridotites, from global mid-ocean ridges [110] (inset, isotherms close to ridges based on half-space cooling model (solid lines of 600, 800, 1000 and 1200°C [28]) and plate model (dashed lines of 1100 and 1300°C [113]), assuming a Δ*T* of 1350°C, an average plate velocity of 8 cm/year and a thermal diffusivity of 1 mm^2^/s (note that the modeling is not sensitive to plate velocity); in (c), garnet peridotite xenoliths, from Stagno *et al.* [119] (including data for MORB [114], spinel peridotites [118] and abyssal peridotites [116,117]). The shallow mantle at ∼40 to 100 km is mostly of low temperature (e.g. <1200°C) and relatively oxidized state (close to FMQ or NNO), with significant lateral heterogeneities (see text). Fe–FeO, IW buffer (≈ Mo–MoO_2_, MMO); Ni–NiO (NNO) is similar to FMQ (with slight differences depending on pressure and temperature).

At depths of 40–150 km in the mantle, the domains are colder in subduction zones than in the normal mantle and are hotter below mid-ocean ridges than away from ridges [103]. These reflect the general trends in temperature variation (or thermal gradient) in different settings and the variations are present also in the same or similar settings. In continental regions, the temperature of garnet peridotite xenoliths [104,105], estimated by using the widely applied thermo-barometer pairs [106,107], is mostly <1300°C at depths of <200 km and the lateral variation can be >400°C and up to ∼300°C at a similar depths at the global and locality scales, respectively (Fig. 4a). The temperature is relatively low in Archean and Proterozoic cratons, but can be high in Phanerozoic thinned cratons and active zones [104,108]. The xenolith-based temperatures are nearly consistent with those in the analysis of surface heat flux [109], although the latter may be subject to invalid assumptions of a steady-state conductive geotherm and poorly constrained distributions of heat-producing elements in the mantle and crust [104]. In oceanic areas, the temperature of abyssal peridotites from mid-ocean ridges [110], estimated by using the often adopted two-pyroxene thermometry [111], is mostly 850–1250°C (Fig. 4b). Abyssal peridotites have commonly been spinel-bearing (free of garnet) for the specimens found so far [110]. The stability of spinel peridotites is restricted mainly to depths of <70 km [112]: the temperature at such depths below ridges is thus mainly <1250°C, being highly heterogeneous at both global and local scales [110]. The sample-based temperatures offer a basis for the thermal structure in the regions away from ridges and their patterns broadly agree with those of the half-space cooling modeling [28] and the plate modeling [113] (Fig. 4b).

The mantle redox state is also heterogeneous. In the shallow mantle, the zones are often more oxidized in mantle wedges than beneath ridges and plume-related regions can be similarly oxidized to those in mantle wedges [114,115]. These reflect the redox variations over different settings. In oceanic regions, the redox state of MORB is similar to that in the fayalite–magnetite–quartz buffer (FMQ, ≈ NNO), mostly at FMQ–0.5 to FMQ+0.5 [114], but the redox state of abyssal peridotites is from FMQ–4.5 to FMQ+1 [116,117] (Fig. 4c). The contrast may be due to the homogenization of redox heterogeneities during the melting of the mantle, as reflected by the relatively unified *f*O_2_ of MORB. The redox state of the oceanic mantle (probably including the asthenosphere) can be greatly heterogeneous, as observed in abyssal peridotites [116,117]. In continental regions, the redox state of spinel peridotite xenoliths is mostly FMQ–1.5 to FMQ+0.5 in cratons/shields [118] (Fig. 4c), but is primarily FMQ–1 to FMQ+3 in mantle wedges [114]. The redox heterogeneities at different scales are well displayed by garnet peridotite xenoliths (Fig. 4c). The mantle evolves from around FMQ at depths of <70 km (e.g. for spinel peridotite xenoliths) to as low as FMQ–4 at depths exceeding ∼150 km and, at similar depths in a regional locality, the redox variation is 2–4 log units [119]. Consequently, the upper mantle is broadly redox-stratified, but with significant lateral heterogeneities.

## ROLES OF MANTLE HETEROGENEITIES IN PRODUCING ELECTRICAL ANOMALIES

The widespread heterogeneities of petrological constituents, mineral compositions and thermodynamic properties in the upper mantle indicate a framework that differs from the widely assumed ideal scenarios for explaining the mantle electrical structure in previous studies. The impacts of the heterogeneities on geophysically imaged electrical anomalies are examined (melt conductivity is discussed above), based on measured data from experiments at high pressures and temperatures and by using impedance spectroscopy.

### Electrical conductivity of pyroxenites

Pyroxenites essentially consist of minerals in the pyroxene group and are classified into orthopyroxenites, clinopyroxenites and websterites, including the special megacryst orthopyroxene or clinopyroxene (i.e. augite). The relatively Fe- and/or water-rich nature of pyroxenes in mantle pyroxenites, as already briefly discussed above, may enhance electrical conductivity.

**Figure 5.**
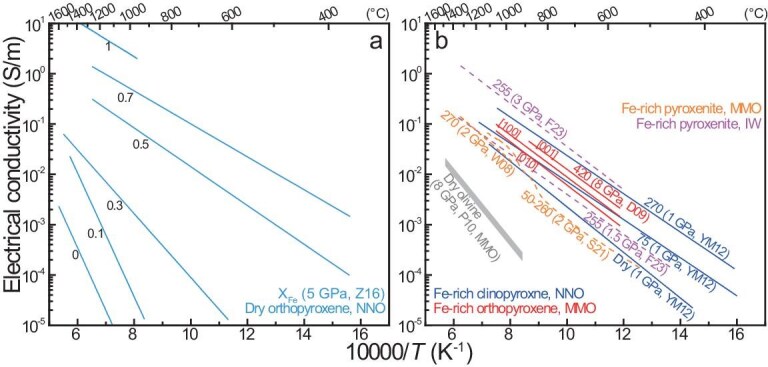
Electrical conductivity of pyroxenites. (a) Fe effects on orthopyroxene and (b) conductivity of Fe-rich pyroxenites. Labels are *X*_Fe_ (= Fe/(Fe + Mg), in molar ratios) in (a) and water content (wt. ppm H_2_O) in (b), with other details. In (b), solid lines denote pyroxene megacrysts (clinopyroxene, 8.31 wt.% FeO; orthopyroxene, 9.12 wt.% FeO) and dashed lines denote bulk pyroxenites. Under fixed conditions, conductivity increases with increasing Fe or water content (see text). The electrical anisotropy of H-bearing pyroxenes is weak [35,124] and the conductivity of orthopyroxene along three axes [124] is shown. The dry olivine conductivity of Poe *et al.* [34] (Fig. 1) is shown. Data sources: W08, Wang *et al.* [126]; D09, Dai and Karato [124]; YM12, Yang and McCammon [125]; Z16, Zhang and Yoshino [123]; S21, Saxena *et al.* [128] (bulk water content of 50–260 wt. ppm H_2_O); F23, Ferrand and Chin [127].

In dry pyroxenes, the conductivity is substantially enhanced at higher Fe contents, as observed for clinopyroxene [120,121] and orthopyroxene [122,123]. Figure 5a shows a typical example of the effects of Fe on the conductivity of dry orthopyroxene [123]. In general, the conductivity increases and activation enthalpy decreases with increasing Fe content under comparable conditions. Figure 5b shows a compilation of the conductivity data of different pyroxenite samples. In general, the conductivity increases with water content given other conditions, and the influences of water at low-to-moderate contents are significant at high temperatures of ≤1200°C, as seen for orthopyroxene at 8 GPa [124] and clinopyroxene at 1 GPa [125]. This is quite different from the possible effect of water on the conductivity of olivine that is not profound above ∼1000°C for a content of up to ∼500 wt. ppm H_2_O (as will be discussed below). The electrical anisotropy of both H-bearing clinopyroxene and orthopyroxene, for water contents of up to ∼700 wt. ppm H_2_O, is not obvious [35,124], resembling olivine with similar water contents (Fig. 1). For clinopyroxene with ∼8.31 wt.% FeO and 75–270 wt. ppm H_2_O and orthopyroxene with ∼9.12 wt.% FeO and 400 wt. ppm H_2_O, the conductivity can be >0.1 S/m at >1000°C (Fig. 5b). Similarly high conductivities have also been determined for pyroxenites containing both clinopyroxene and orthopyroxene, with similar contents of both Fe and water ([126–128]: Fig. 4b). For pyroxenites with higher FeO and/or water contents in the constituting pyroxenes, e.g. up to ∼15 wt.% FeO and >1000 wt. ppm H_2_O in clinopyroxene (Fig. 3b and c) and up to >20 wt.% FeO in orthopyroxene (see above), the conductivity is expected to be even greater, owing to the significant roles of Fe and water in enhancing electrical conduction.

The experimental data (Fig. 5) exhibit that, at a fixed temperature, the conductivity of pyroxenites with a moderate Fe content and a low-to-moderate water content could be orders of magnitude higher than that of dry olivine reported by Poe *et al.* [34]. Thus, Fe- and/or water-rich pyroxenites are more conductive than peridotites under similar conditions. The occurrences of pyroxenites in peridotites in the mantle, mostly in connected forms (e.g. veins, dykes and/or layers, as already noted), can be treated as parallel circuits for bulk electrical conduction. In this case, the bulk conductivity can be easily raised to ∼0.01–0.1 S/m or even greater under mantle conditions if the fraction of pyroxenites is <1 vol.% to several % vol.% (within the range of petrological estimates from natural samples) [125,127]. This is through modeling with either the parallel solution or the Hashin–Shtrikman upper bound that is commonly applied to a sample mixture (especially when the conductive phase is interconnected). The conductivity is high along the direction(s) of the connected pyroxenites, but is not enhanced along other directions (e.g. pyroxenites not connected), being able to cause electrical anisotropy. The needed pyroxenite fraction for high conductivity under given conditions (e.g. temperature) can be even less when the constitutive pyroxenes are more Fe- and/or water-rich.

### Electrical conductivity of eclogites

Eclogites chiefly consist of omphacite and garnet, which are key to controlling bulk conductivity. Omphacite is rich in Na compared with clinopyroxene in peridotites and pyroxenites. However, the conductivity of omphacite is probably not largely affected by Na, as indicated by experimental work on samples with different Na contents [129]. This differs from silicate melts, whose conductivity is largely controlled by the transfer of Na (and/or other alkalis) [55]. The relatively Fe- and/or water-rich nature of omphacite and garnet in eclogites, as noted above, may enhance electrical conductivity.

**Figure 6.**
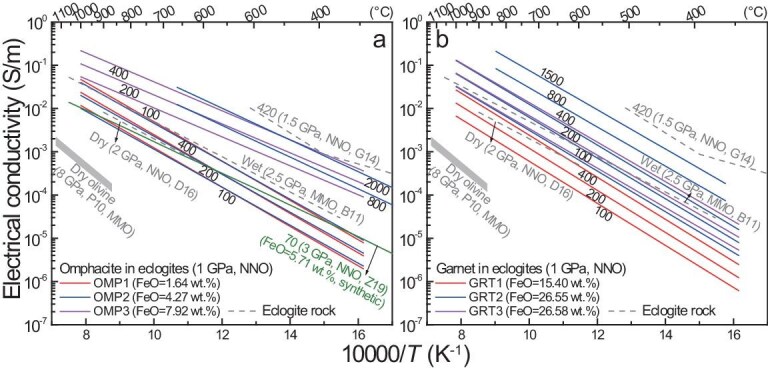
Electrical conductivity of eclogites. (a) Omphacite and (b) garnet. Labels are for water content (wt. ppm H_2_O) and other details. Data are modeled for samples with 85–2000 wt. ppm H_2_O in omphacite and 40–1460 wt. ppm H_2_O in garnet based on laboratory studies of similar sample water contents. A synthetic omphacite is shown in (a) and the grey dashed lines in (a) and (b) indicate bulk eclogites. Under the given conditions, theconductivity increases with Fe or water content (see text). The dry olivine conductivity of Poe *et al.* [34] (Fig. 1) is shown. The water content of eclogite in G14 is from constituting omphacite and garnet, and the water content of eclogite in B11 was not reported but should be ∼200–400 wt. ppm H_2_O based on samples from similar regions. Data sources: omphacite and garnet at 1 GPa, Liu *et al.* [129,130] and Liu and Yang [131]; B11, Bagdassarov *et al.* [133]; G14, Guo *et al.* [134]; D16, Dai *et al.* [135]; Z19, Zhang *et al.* [132].

The conductivities of H-bearing natural [129–131] and synthetic [132] omphacite are given in Fig. 6a. Under fixed conditions, the conductivity of omphacite (natural samples) increases with water content (from ∼100 to 2000 wt. ppm H_2_O). Given other conditions, the conductivity is similar for two samples with ∼1.6 and 4.3 wt.% FeO, but is ∼10 times greater for a sample with ∼7.9 wt.% FeO, of which the activation enthalpy is lower than in the former two Fe-less samples (∼65 versus 85 kJ/mol, as reflected by slopes of data). This implies that the Fe effect on the omphacite conductivity is not profound over the range of 1.6–4.3 wt.% FeO but is significant at a content of ∼7.9 wt.% FeO. Under similar conditions, the conductivity of a synthetic omphacite with ∼70 wt. ppm H_2_O and 5.7 wt.% FeO is in line with that of the natural samples. The conductivities of H-bearing garnet in eclogites [129–131] are plotted in Fig. 6b (excluding data for garnet that are not typical of eclogites). Given other conditions, the conductivity of garnet is enhanced by both Fe (∼15.4 to 26.6 wt.% FeO) and water (100 to 1500 wt. ppm H_2_O). The activation enthalpy is, however, broadly the same (∼90 kJ/mol) over the wide content ranges of both Fe and water, differing from omphacite (Fig. 6a). In general, for both omphacite and garnet of 100–400 wt. ppm H_2_O, the conductivity can be >0.01 to >0.1 S/m at >1000°C and can be even higher when the sample Fe and/or water contents are greater (Fig. 6). The modeled conductivity of bulk eclogites based on their constituting omphacite and garnet can be similarly high at >1000°C [129]. Similar high conductivities have been measured for bulk eclogites with similar Fe and water contents ([133–135]: Fig. 6b).

The measured data (Fig. 6) show that, given low-to-moderate Fe and water contents in the constituting omphacite and garnet under otherwise similar conditions, the conductivity of eclogites can be orders of magnitude higher than that of dry olivine reported by Poe *et al.* [34]. As such, eclogites are able to be more conductive than peridotites given other conditions. The occurrences of eclogites in peridotites in the mantle, mostly in connected forms (e.g. veins, dykes and/or layers, as noted above), can be reasonably considered as parallel circuits for bulk electrical conduction (resembling pyroxenites). The regional presence of eclogites, e.g. a few to ∼10 vol.% (within the range of petrological estimates from natural samples), can raise the bulk conductivity to 0.01–0.1 S/m or even greater (when modeling with the parallel solution or the Hashin–Shtrikman upper bound) [129–131]. Therefore, electrical anisotropy can be produced by the spatial distribution of eclogites relative to peridotites in the mantle. We want to emphasize that the volume estimates are made by extrapolating the conductivity measured at <800°C: it is unclear whether the extrapolation of omphacite is affected by its phase transition from *P*2/*n* to *C*2/*c* at ∼750–900°C that differs from other mantle minerals [136,137].

### Electrical conductivity of hydrous minerals

**Figure 7.**
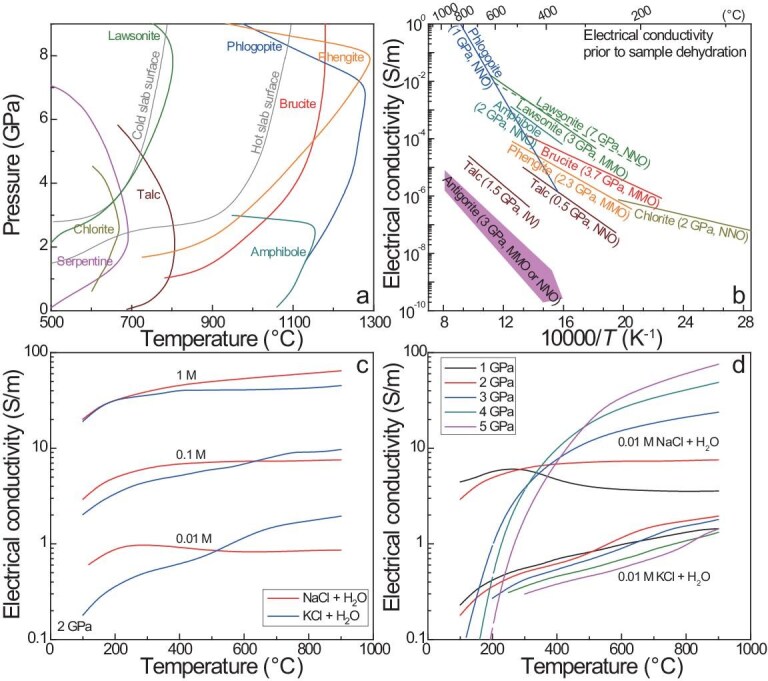
Electrical conductivity of hydrous minerals. (a, b) Stability fields + conductivity of minerals; (c, d) conductivity of aqueous fluids. Hydrous minerals are usually resistive in the stability fields, except phlogopite, and the conductivity of aqueous fluids increases with the brine fraction (M refers to mol NaCl (or KCl) per liter). Data sources: (a) stability fields, mostly of Schmidt and Poli [82] and Frost [78]; slab geotherms, D80 model of Syracuse *et al.* [103]; (b) phlogopite (geometric mean along three fundamental directions), Li *et al.* [93]; antigorite, Reynard *et al.* [138]; talc, Wang and Karato [139] and Hao *et al.* [140]; brucite, Guo and Yoshino [146]; lawsonite, Mathilake *et al.* [144] and Pommier *et al.* [145]; chlorite, Manthilake *et al.* [143]; amphibole, Hu *et al.* [142]; phengite, Chen *et al.* [141]; (c, d) NaCl + H_2_O, Guo and Keppler [149]; KCl + H_2_O, Vlasov and Keppler [150].

Many hydrous minerals may occur in the shallow mantle, e.g. amphibole, chlorite and phlogopite. Each mineral is stable over limited pressures and temperature ranges, and the stability fields are provided in Fig. 7a (note that some of these fields are for simple or ideal systems). Possible effects of hydrous minerals on the mantle electrical structure are 2-fold: (i) the high water contents (at wt.% levels) may make them highly conductive and (ii) aqueous fluids, by their dehydration, may enhance bulk conductivity.

The conductivities of hydrous minerals, including those of phlogopite [93,94], antigorite [138], talc [139,140], phengite [141], amphibole, [142], chlorite [143], lawsonite [144,145] and brucite [146], prior to dehydration are plotted in Fig. 7b. Except for phlogopite, the hydrous minerals (especially antigorite) are resistive at <600°C, e.g. <10^−4^ S/m for most of them (excluding amphibole and lawsonite), and <0.01 S/m for all of them. Under comparable conditions, the conductivity of phlogopite increases with F content. For phlogopite with a moderate F content of 2.7 wt.%, the conductivity can be ∼0.01 S/m at 600°C and 1 S/m at 900°C (Fig. 7b). The high conductivity of phlogopite is due to F and the activation enthalpy is 134–204 kJ/mol. For other hydrous minerals, the activation enthalpy is often <75 kJ/mol and the very low conductivity implies that the electrical conduction is unlikely to be due to the high water content, differing from the H conduction in nominally anhydrous minerals. Within the stability fields, most hydrous minerals are unable to cause a high conductivity of >0.01 S/m in the mantle (Fig. 7). The exception is phlogopite, whose stability field is wide: when occurring as connected forms (e.g. veins), a minor fraction of phlogopite in peridotites, e.g. <0.5 to several vol.% (within the range of petrological estimates from natural samples), can raise the bulk conductivity to 0.01–0.1 S/m or higher (and the electrical anisotropy) at 900–1200°C (limited mostly to depths of <250 km).

Aqueous fluids are produced by the dehydration of hydrous minerals, as well as the degassing of externally derived magmas [147]. Water is a major component in the fluids and brine (NaCl or KCl) dissolution largely enhances the conductivity. Conductivity data are plotted in Fig. 7c and d for NaCl–H_2_O [148,149] and KCl–H_2_O [150]. In general, the conductivity increases with the brine fraction and a positive effect of temperature is obvious at >500°C. Given other conditions, the conductivity of NaCl–H_2_O differs variably from that of KCl–H_2_O, depending on the pressure, temperature and brine fractions [148–150]. The conductivity of both NaCl–H_2_O and KCl–H_2_O is high, at ∼50 S/m for a brine fraction of 1 M at 600°C (2 GPa). This is due to the high mobility of the dissociated Cl^−^, Na^+^ and K^+^. The high conductivity suggests that, in subduction zones, ∼1 vol.% aqueous fluids may increase the bulk conductivity to 0.1–1 S/m [148–150], when modeled by using the parallel solution or the Hashin–Shtrikman upper bound. When the wetting property [151] and grain-size effect [65] are considered, the required fluid fraction for a fixed conductivity would be greater, e.g. by a factor of 5–10 or higher (see melt effects above). Aqueous fluids are produced in subducting slabs, in which conductive signals have, however, not been geophysically imaged [152,153]. This may imply that: (i) the fraction of fluids in subducting slabs is low and/or (ii) fluids, once generated, immediately escape upward from the system. In the case of upward flow due to buoyancy in either a pervasive or a channelized mode, however, fluids are unlikely to produce zones of significant lateral electrical anisotropy.

### Electrical conductivity of non-silicate assemblages

Of the non-silicate assemblages that are present in the upper mantle, graphite and sulfides are two typical components that may influence the mantle electrical structure. This is due to the highly conductive behaviors of the two materials, with the conductivity of both in the order of ∼10^5^ S/m, which is nearly temperature-independent [128,154]. In theory, the presence of a very minor fraction (e.g. far less than 0.1 vol.% in theory) of graphite or sulfides in a peridotite matrix may greatly increase the bulk conductivity.

**Figure 8.**
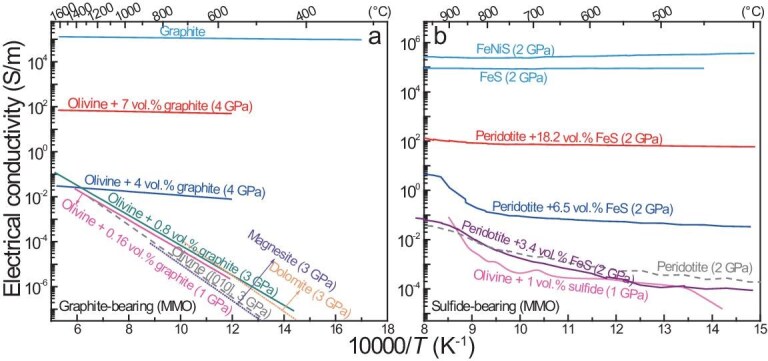
Electrical conductivity of non-silicate assemblages. (a) Graphite and graphite-bearing olivine and (b) sulfide and sulfide-bearing olivine/peridotite. Bulk conductivity increases with the graphite or sulfide fraction. Data in (a) are from Zhang and Yoshino [154], with a dashed grey line for dry olivine ([010]), and data in (b) are from Saxena *et al.* [128] and Watson *et al.* [155], with a dashed grey line for a dunite (95 vol.% olivine, 50–80 wt. ppm H_2_O). Dotted lines in (a) denote magnesite [157] and dolomite [158] at 3 GPa. Note that, in Saxena *et al.* [128], Re heater and Mo/Fe electrodes were used (Re–ReO_2_ was 5–7 log units more oxidized than MMO) and a middle (oxidized) state around NNO was possible.

The conductivities of graphite-bearing and sulfide-bearing olivine and peridotite are plotted in Fig. 8a and b, respectively. In graphite-bearing samples, the bulk conductivity increases with graphite fractions of ∼0.2 to 7 vol.% and the yielded activation enthalpy is insensitive to temperature with higher graphite fractions of 4–7 vol.% [154]. Given other conditions beyond ∼800°C, the conductivity is similar between graphite-free olivine and graphite-bearing olivine with 0.2–0.8 vol.% of graphite, indicating that the conductivity is barely enhanced by graphite over those fractions (Fig. 8a). In sulfide-bearing samples, the bulk conductivity increases with sulfide fractions of ∼1 to 18.2 vol.% [128,155]. Under given conditions (>500°C), the conductivity is close between the sulfide-free peridotite and sulfide-bearing samples with 1–3.4 vol.% of sulfides, implying that the conductivity is barely enhanced by sulfide over those fractions (Fig. 8b). This is mainly due to the fact that, for fractions of up to ∼0.8 vol.% of graphite and ∼3.4 vol.% of sulfides, both are unable to form connected networks under mantle conditions [128,154]. This differs from pyroxenites, eclogites and phlogopite, which can occur in connected forms (veins, dykes and/or layers) in peridotites, for which the bulk conductivity can be reasonably modeled by using the parallel solution or the Hashin–Shtrikman upper bound.

To enhance the bulk conductivity of olivine or peridotite, three prerequisites should be met for graphite or sulfides: (i) they must occur as connected networks, e.g. grain boundary films or veins; (ii) the volume fractions must be large enough and also consistent with mantle abundances; and (iii) they must be stable under mantle pressure, temperature and redox conditions. In mantle xenoliths, both graphite and sulfides are commonly dispersed, but not connected (as noted above). In rare cases, graphite films are seen in some mantle xenoliths, but they are stable only to ∼600–900°C, indicating their formation during sample decompression or cooling [156]. The bulk abundance is only ∼10–30 wt. ppm C and 140 ± 30 wt. ppm S in the depleted mantle [61] and the estimated fractions of both graphite and sulfides in mantle xenoliths are very low, e.g. <0.1 vol.% (as mentioned above: note that relatively graphite-rich xenoliths have been found occasionally, but the graphite in them is commonly dispersed [88]). Finally, graphite is stable only under reduced conditions, e.g. with a redox state of greater than ∼1.2 log units below FMQ, and it is unstable in most of the oxidized shallow mantle, including the asthenosphere [114], although sulfides can be stable in the upper mantle. Considering all these aspects, graphite and sulfides are unlikely to be the effective candidates that may account for the imaged mantle electrical anomalies. Regarding carbonate minerals in the upper mantle, e.g. dolomite and magnesite, they are, if present, usually not in connected forms in peridotites [91] and, especially, they are less conductive [157,158] (Fig. 8a). Therefore, carbonate minerals cannot produce high conductivity in the shallow mantle.

### Electrical conductivity of olivine owing to redox effects

The conductivity of olivine due to H conduction itself is discussed above. The data in most available reports are broadly consistent with each other, except for a few samples (Fig. 1). The bulk conductivity of H-bearing olivine should include contributions from both small-polaron conduction and H conduction, especially at high temperatures, when the roles of small-polaron conduction can be significant.

**Figure 9.**
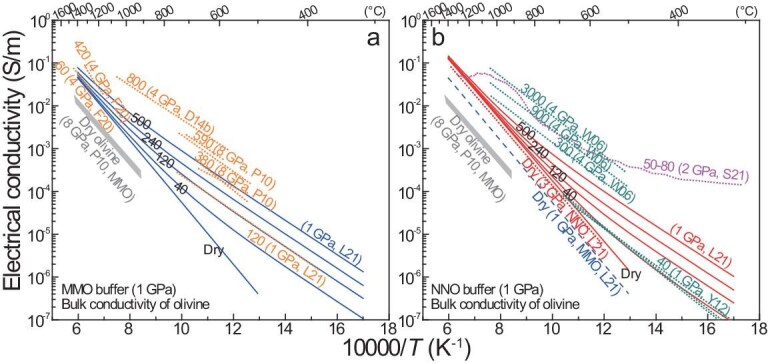
Bulk electrical conductivity of olivine. (a) MMO buffer and (b) NNO buffer. Water contents and other details are labeled. Solid lines indicate the modeled bulk conductivity of olivine with different water contents (from runs using relatively sealed double capsules for accurate redox and dimension controls: [37]). Dotted lines in (a) and (b) indicate the measured data of olivine (+ a dunite consisting of 95 vol.% olivine, labeled S21: see Fig. 8b) and the dashed line in (b) indicates the conductivity of dry olivine in (a) (by the same assembly design and analytical method). Conductivity data of F20 are for two samples with less dehydration. Conductivity of dry olivine in Poe *et al.* [34] (Fig. 1) is shown. Water contents are based on Bell *et al.* [39] and Mg# is ∼90 for all olivines. Data sources: W06, Wang *et al.* [18]; P10, Poe *et al.* [34]; D14b, Dai and Karato [36]; Y12, Yang [35]; F20, Fei *et al.* [159]; L21, Liu *et al.* [37]; S21, Saxena *et al.* [128].

By using relatively sealed boron–nitride metal double capsules for accurate redox and dimension controls, the conductivity of dry and H-bearing olivines under well-controlled conditions has been measured [37]. This enabled modeling of the bulk conductivity of olivine with 0–500 wt. ppm H_2_O by considering the maximum water solubility under shallow mantle conditions (see above). The results are shown in Fig. 9a and b, including the data in typical work [18,34–37,159]. For runs buffered by either MMO or NNO, the conductivity is obviously enhanced by water only at low temperatures (e.g. <700°C) and the enhancement is not vital at >1000°C. For the MMO buffer, the modeled bulk conductivity versus water content agrees with the data on olivine, at ∼60–800 wt. ppm H_2_O in Fei *et al.* [159], Liu *et al.* [37], Poe *et al.* [34] and Dai and Karato [36] (Fig. 9a). For the NNO buffer, the modeled bulk conductivity versus water content agrees with the data on olivine, at ∼40–3000 in Yang [35] and Wang *et al.* [18] (Fig. 9b), the latter of which are slightly greater (probably due to the relatively large uncertainties: see their original figure 2). For dry olivine buffered by MMO, the data of Liu *et al.* [37] at 1 GPa are ∼0.5 log units greater relative to Poe *et al.* [34] at 8 GPa (Fig. 9a). This can be explained by the pressure effect, e.g. conductivity decreases by 0.1–0.2 log units for a pressure increase of 2–3 GPa (as noted above).

Under given conditions, the conductivity is ∼0.5 log units higher in dry olivine buffered by NNO rather than by MMO (Fig. 9b). This is due to the increase in Fe^3+^ at higher *f*O_2_ that facilitates small-polaron conduction. The exponent ∼0.1 for *f*O_2_ [37] (i.e. given other conditions, the conductivity is proportional to $f{\mathrm{O}}_{\mathrm{2}}^{{\mathrm{\sim 0}}{\mathrm{.1}}}$) resembles the inferred 2/11 or 1/6 for stoichiometric olivine of ideal defect populations [160]. The bulk conductivity of olivine in the mantle is thus redox-dependent, when considering the trivial effects of water for a content of up to ∼500 wt. ppm H_2_O (or <100 wt. ppm H_2_O in most natural samples) at >1000°C. For a temperature of 1000–1300°C, the conductivity is mostly <0.03 S/m at 1 GPa with the MMO buffer, but is ∼0.01–0.1 S/m at 1–3 GPa with the NNO buffer (Fig. 9). In zones of low temperatures (<1000°C) in the shallow mantle, the conductivity is <0.01 S/m, even if the redox state around the NNO prevails. In the deep shallow mantle at depths beyond ∼150 km, a reduced state similar to MMO is possible (Fig. 4c) and the conductivity is mostly <0.01 S/m after correcting for the pressure effect. Thus, the conductivity of olivine in the upper mantle can be 0.01–0.1 S/m given a proper high temperature (1000–1300°C) and oxidized state (∼NNO): if such conditions are maintained as broadly continuous along a certain direction, electrical anisotropy due to olivine is produced by the spatial patterns of thermodynamic heterogeneities. The modeled high conductivity of olivine under oxidized conditions agrees with the report for a dunite consisting nearly entirely of olivine (50–80 wt. ppm H_2_O) by Saxena *et al.* [128] (Fig. 9b). Note that the conductivity of pyroxenites or eclogites is also enhanced under more oxidized conditions (for small-polaron conduction), but the data under both reduced and oxidized conditions are quite high (Figs 5 and 6).

### Why is the shallow upper mantle not globally conductive?

As discussed in previous sections, four candidates are possible for mantle electrical anomalies: (i) olivine under an oxidized state (around NNO), (ii) lithologies of pyroxenites, eclogites and phlogopite-bearing assemblages (e.g. lithologies for simplicity), (iii) partial melt and (iv) aqueous fluids. Candidates (i) and (ii) are widespread, candidate (iii) can be present beneath ridges and hot spots and in mantle wedges, and candidate (iv) may occur in mantle wedges. In this context, the shallow mantle may be extensively conductive, but this is obviously not the case when imaged by geophysical studies.

Several factors are important for the four candidates. (i) Suitable temperatures: the conductivities of olivine, lithologies and partial melt are all temperature-sensitive, differing from fluids (Figs 2, 5–7 and 9). High conductivity is hard to produce at temperatures of <1000°C. The temperature is mostly 600–1300°C at depths of <200 km in the continental mantle (Fig. 4a, xenolith data), 850–1250°C at depths of <70 km below ridges (Fig. 4b, abyssal peridotite estimates) and 600–1300°C at depths of <90 km in the oceanic mantle away from ridges (Fig. 4b, thermal modeling: temperatures of >1300°C are likely in narrow spaces below ridges). (ii) Connected networks: a connected form of a candidate is a prerequisite for contributing to bulk conductivity. Except for the most abundant olivine, globally connected networks are unlikely to be common for any other candidates, though connected veins, dykes and/or layers are possible in regional zones, whereas, for olivine, a conductivity of 0.01–0.1 S/m requires temperatures of 1000–1300°C and a redox state similar to NNO (Fig. 9), for which the global continuity is unlikely to be due to the heterogeneities (Fig. 4). (iii) Rational fractions: the fraction of a candidate in a matrix, and if the amount is reasonable, is key to causing high conductivity. This is critical for all the candidates except olivine. While the fractions of lithologies in connected direction(s), inferred from mantle samples, could produce high conductivity, they may not be valid along other directions. For melts/fluids, their required fractions for high conductivity are greater than previous estimates for ideal systems.

**Figure 10.**
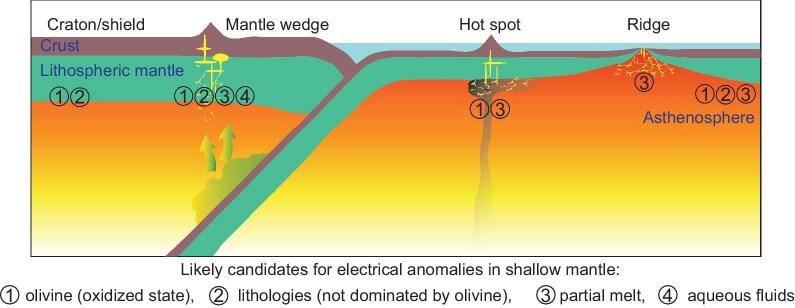
Sketch of conductive candidates in the upper mantle. Four candidates are shown, including olivine at the oxidized state, lithologies of pyroxenites, eclogites and phlogopite-bearing assemblages, partial melt and aqueous fluids. Electrical anomalies in different areas are due to different candidates. Conductivity of up to ∼1 S/m or higher below ridges is more likely due to locally enriched melt and high temperature (though olivine of oxidized state also contributes). Complexity in mantle wedges is that the transfer of melt or aqueous fluids can metasomatize peridotites and produce conductive lithologies and oxidized states, making the matrix relatively conductive. The dominant candidate for a specific domain may be inferred from its exact conductivity, lithologies, redox state and thermal structure.

These factors make the shallow mantle unlikely to be globally conductive. The four candidates are not independent: fluids can trigger melting and reactions of fluids or melts with peridotites lead to lithologies (e.g. metasomatism). The macroscale heterogeneities of constituents, compositions and thermodynamic properties can thus produce electrical anomalies. The active candidates differ in different settings: (i) olivine at the oxidized state or lithologies for cratons/shields, (ii) olivine at the oxidized state, lithologies, melts or fluids for mantle wedges, (iii) olivine at the oxidized state or melts for plume-related areas, (iv) melts for ridges and (v) olivine at the oxidized state, lithologies or melts for oceanic asthenosphere (Fig. 10). It is likely that, in certain regions, several of the candidates are mutually active. Of particular interest is to distinguish solid candidates (olivine at the oxidized state and lithologies) from liquid candidates (melts and fluids). A possible way is to map whether the electrical structure is stable. This is because melts or fluids of the needed high fractions, as noted above, would be mostly driven out of the system by efficient compaction [20,161] and react with peridotites [24], being unstable over geological scales. This indicates that, different from the solid candidates, the regionally abundant melts or aqueous fluids may be more capable of producing a transient rather than stable structure of high conductivity.

## CONCLUDING REMARKS AND PERSPECTIVES

The origin of electrical anomalies in the upper mantle is critical to understanding its dynamics and evolution. A correct interpretation of the electrical anomalies requires the integrated constraints from petrological and geochemical studies, geophysical mappings and mineral physics experiments. Examining mantle samples shows heterogeneities of the upper mantle regarding its constituents (mineral assemblages, melts and fluids), compositions (e.g. Fe and H_2_O content) and thermodynamic properties (temperature and redox state). Experimental studies have identified four conductive candidates that are crucial for the electrical anomalies: olivine at the oxidized state, lithologies (pyroxenites, eclogites and phlogopite-bearing assemblages), partial melt and aqueous fluids.

At mantle temperatures of >1000°C, the conductivity of olivine is barely enhanced by water for a potential content in the mantle, but is greater under more oxidized conditions (as prevailing in the shallow mantle). In contrast, high conductivity is caused by high Fe and/or water contents for pyroxenites and eclogites and by high F content for phlogopite if they are present mostly as connected veins, dykes and/or layers. The roles of melts are possible in zones below ridges and hot spots and in mantle wedges, and maybe also in the oceanic asthenosphere, and the roles of aqueous fluids are mainly restricted to mantle wedges. The effects of melts and aqueous fluids on bulk conductivity are controlled by their connectivity and wetting properties, and the fractions required for a fixed conductivity under given conditions are greater than previously estimated. Hydrous minerals within their stability fields, except phlogopite, and non-silicate minerals of graphite, sulfides and carbonates are unlikely to lead to electrical anomalies. The high conductivity of olivine under an oxidized state and lithologies lays a new framework for mantle electrical properties. The heterogeneous temperature and redox state of the upper mantle affect the conductivity of olivine and other candidates (including the stability of melt), contributing to the heterogeneous electrical structure. In regions in which melts and fluids must be invoked for high conductivity, a conductive framework of olivine at the oxidized state and lithologies could reduce the required melt fractions (but mostly still at vol.% levels); however, if the temperature is <1000°C, the melting status of melts cannot be always maintained. In the upper mantle, the composition of a melt or fluid would evolve upon transferring upward through a reaction with wall rocks [162]. Such changes could produce subtle changes in the material conductivity, but the general discussions above are not affected (note that the conductivity of a melt/fluid is usually very high and conductivity experiments are usually performed on a sample in equilibrium).

The macroscale heterogeneities of constituents, compositions and thermodynamic properties arise from a series of processes, e.g. early differentiation, thermal conduction, subduction, convection and metasomatism. As such, the electrical anomalies resolved by geophysical studies are linked to the petrological and geochemical evolution of the upper mantle. Low-conductivity (e.g. <0.01 S/m) domains reflect the relatively cold (e.g. <1000°C) and/or reduced conditions of an olivine-dominant matrix. However, the interpretation of many conductive zones remains to be studied, particularly when several candidates are likely for a certain domain (Fig. 10). Further studies from the following aspects would help to better understand the electrical anomalies and the relevant properties: (i) characteristic lengths of petrological and geochemical heterogeneities that enable the sizes of conductive regions to be constrained; (ii) accurate thermal structures that determine the exact candidate for electrical anomalies in many regions (including whether melts occur); (iii) improved resolution of electromagnetic imaging so that the fraction of a non-olivine candidate at a given temperature is quantified; (iv) long-period monitoring of conductivity signals for a stable-versus-transient structure for distinguishing the possible role of a solid-versus-liquid candidate; (v) threshold value of partial melt that is mechanically stable and geologically meaningful, and whether large-volume melts can be trapped, which is vital for the melt candidate; (vi) conductivity experiments under well-controlled conditions of pressure, temperature, redox state and sample chemistry, and only accurately measured data can be used to evaluate a conductive candidate and explain the electrical anomalies. The combination of geological, geophysical and geochemical studies and mineral physics experiments should be effective for better understanding the origin of mantle electrical anomalies, which can be facilitated by conducting joint surveys in some typical tectonic domains. The application of machine learning (and artificial intelligence) to available data may shed new light on this issue, but data must first be filtered for quality control and mantle heterogeneities must be taken into account. This is particularly critical for the electrical structure in oceanic areas away from ridges, which is key to documenting the origin of the asthenosphere and plate tectonics.
